# Ferulic acid combined with aspirin demonstrates chemopreventive potential towards pancreatic cancer when delivered using chitosan-coated solid-lipid nanoparticles

**DOI:** 10.1186/s13578-015-0041-y

**Published:** 2015-08-21

**Authors:** Arvind Thakkar, Sushma Chenreddy, Jeffrey Wang, Sunil Prabhu

**Affiliations:** Department of Pharmaceutical Sciences, College of Pharmacy, Western University of Health Sciences, 309 E Second Street, Pomona, CA 91766 USA

**Keywords:** Chemoprevention, Pancreatic cancer, Ferulic acid, Aspirin, Chitosan, Solid lipid nanoparticles

## Abstract

**Background:**

The overall goal of this study was to demonstrate potential chemopreventive effects of ferulic acid (FA), an antioxidant, combined with aspirin (ASP), a commonly used anti-inflammatory drug for pancreatic cancer chemoprevention, using a novel chitosan-coated solid lipid nanoparticles (c-SLN) drug delivery system encapsulating FA and ASP.

**Results:**

Our formulation optimization results showed that c-SLNs of FA and ASP exhibited appropriate initial particle sizes in range of 183 ± 46 and 229 ± 67 nm, encapsulation efficiency of 80 and 78 %, and zeta potential of 39.1 and 50.3 mV, respectively. In vitro studies were conducted to measure growth inhibition and degree of apoptotic cell death induced by either FA or ASP alone or in combination. Cell viability studies demonstrated combinations of low doses of free FA (200 µM) and ASP (1 mM) significantly reduced cell viability by 45 and 60 % in human pancreatic cancer cells MIA PaCa-2 and Panc-1, respectively. However, when encapsulated within c-SLNs, a 5- and 40-fold decreases in dose of FA (40 µM) and ASP (25 µM) was observed which was significant. Furthermore, increased apoptosis of 35 and 31 % was observed in MIA PaCa-2 and Panc-1 cells, respectively. In vivo studies using oral administration of combinations of 75 and 25 mg/kg of FA and ASP c-SLNs to MIA PaCa-2 pancreatic tumor xenograft mice model suppressed the growth of the tumor by 45 % compared to control, although this was not statistically significant. In addition, the immunohistochemical analysis of tumor tissue showed significant decrease in expression of proliferation proteins PCNA and MKI67, and also increased expression of apoptotic proteins p-RB, p21, and p-ERK1/2 indicating the pro-apoptotic role of the regimen.

**Conclusion:**

Combination of FA and ASP delivered via a novel nanotechnology-based c-SLN formulation demonstrates potential for pancreatic cancer chemoprevention and could be a promising area for future studies.

## Background

Cancer of the pancreas is the fourth leading cause of cancer in the US affecting about 44,000 Americans each year. It has become increasingly apparent that despite more than 20 years of intensive research on treatment, the survival rate continues to be a dismal <5 % within 5 years of diagnosis [1]. Current treatment modalities are largely ineffective, thus bringing to the forefront the development of alternative strategies to tackle this disease [2]. A viable strategy could be the use of chemopreventive agents to suppress, delay or even reverse the onset of the disease [3–5]. Our group has been interested in the use of novel chemopreventive agents and their combinations for pancreatic cancer prevention. Recently, we have also demonstrated the importance of delivering these agents using novel nanotechnology-based drug delivery systems wherein high efficacy has been achieved at very low doses [3, 6–9].

Pancreatic carcinoma arises from a heterogeneous molecular pathogenesis involving several oncogenic pathways and defined genetic mutations. In the last few years, however, oxidative stress and inflammation have emerged as important risk factors in pancreatic carcinogenesis [10–13]. The reports on the role of reactive oxygen species (ROS) in tumor initiation have proved that oxidative stress acts as a DNA-damaging agent, effectively increasing the mutation rate within cells and thus promoting oncogenic transformation [14]. Additionally, oxidative stress acts by further recruiting inflammatory cells to the site of damage and producing more reactive species. This sustained inflammatory/oxidative environment leads to a loop effect, which can damage healthy neighboring epithelial and stromal cells and over a prolonged time period lead to carcinogenesis [15]. Based on this information, the hypothesis of this study was to combine chemopreventive agents ferulic acid (FA) and aspirin (ASP) encapsulated in c-SLNs for the synergistic chemoprevention of pancreatic cancer.

FA is one of the most abundant antioxidants found in plants. FA has a high antioxidant potential due to its resonance-stabilized phenoxyl radical structure, allowing it to be an effective scavenger of free radicals to show its anti-cancer effects [16]. Recently many researchers have focused their attention on the anti-cancer activity of FA on skin, colon, liver and breast cancers [17–19]. However, no other group has investigated its potential in pancreatic cancer chemoprevention. Non-steroidal anti-inflammatory drugs (NSAIDs) such as ASP are currently the best studied chemopreventive agents for many cancers, and have been demonstrated to modulate the NF-κB pathway [20]. Clinical studies associated with the long term use of ASP for pancreatic cancer prevention, however, have met with mixed results thus far [21–23]. Given these conflicting reports on the use of ASP in pancreatic cancer but, simultaneously, realizing the proven benefits of ASP as a chemopreventive in cancer, it reaffirms the need for further study of this drug in pancreatic cancer prevention.

Solid-lipid nanoparticles (SLNs) were developed over a decade ago but have never before been considered extensively for chemoprevention of pancreatic cancer. For the current study, we are proposing a novel SLN delivery system coated with chitosan (c-SLN). Our previous research in chitosan-based drug delivery systems combined with the current research on SLNs enables us to design an effective hybrid system for chemoprevention. Chitosan is a nontoxic, biodegradable and biocompatible polysaccharide derived from the shells of crustaceans, with proven in vivo safety profile [24, 25]. c-SLNs are biodegradable, bioadhesive and have permeation enhancing properties thus acting as promising vehicles for oral drug delivery with wide range of pharmaceutical applications [26]. The primary amino group in chitosan offers some special properties such as water-solubility, hemocompatibility, and cationic groups which could react with a big number of anions or other negatively charged molecules [27]. And also based on the cationic property, chitosan-based nanoparticles exhibit a mucoadhesive feature because of their positive charge, thereby capable of prolonging their residence time in the negatively charged epithelia in small intestine [28], thus increasing the drug concentration at the site of absorption. Moreover, chitosan can mediate the opening of tight junctions between neighboring epithelial cells reversibly, facilitating the paracellular transport of drug molecules ultimately leading to improved bioavailability of the drugs [29]. Thus, c-SLN combines the advantages of SLN with the biological properties of chitosan as a drug delivery vehicle.

In this study, we have used a novel c-SLN technology for the oral delivery of combinations of FA and ASP for pancreatic cancer to evaluate their combined chemopreventive efficacy in two different human pancreatic cancer cells, MIA PaCa-2 and Panc-1. Additionally, a pancreatic tumor xenograft mouse model was used to determine the efficacy of the chemopreventive regimen.

## Results

### Physical characterization of FA and ASP encapsulated c-SLNs

Based on the methodology described earlier [3, 9], c-SLNs of FA and ASP exhibited initial particle sizes in the nanometer range of 183 ± 46 and 229 ± 67, respectively. All the c-SLNs showed optimal particle size with low variability. The c-SLNs exhibited 80 and 78 % encapsulation efficiency of the FA and ASP respectively within the lipid nanoparticles. The zeta potential of FA particles were −8.0 and 39.1 mV before and after coating with chitosan, respectively. The surface charge on ASP nanoparticles were −6.0 and 50.3 mV before and after coating with chitosan, respectively. The polydispersity index (PDI) was 0.25 and 0.19 for FA and ASP c-SLN, respectively (Table 1).

**Table 1:** Particle size and encapsulation efficiency of drug loaded chitosan-solid lipid nanoparticles

Drug	Particle size (nm)	Encapsulation efficiency (%)	Zeta potential (mV)	Polydispersity index (PDI)
Ferulic acid c-SLN	183 ± 46	80	39.1 ± 3.4	0.25 ± 0.06
Aspirin c-SLN	229 ± 67	78	50.3 ± 7.3	0.19 ± 0.05

*c-SLN* chitosan solid lipid nanoparticle

### In vitro evaluation of FA and ASP drug release from c-SLNs

**Fig. 1:**
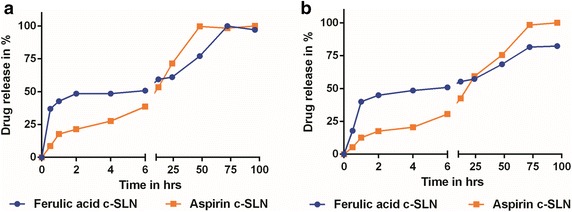
In vitro drug release from chitosan solid lipid nanoparticle (c-SLN). **a** In vitro drug release from c-SLNs over a period of 96 h in phosphate buffered saline (PBS), pH 6.8; and **b** in vitro drug release from c-SLNs over a period of 96 h in acidic medium, pH 1.6. The drug release was analyzed using a HPLC system

The ability of nanoparticles to deliver drugs was examined by determining the drug release in PBS solution (pH 6.8) and acidic conditions (pH 1.6), as shown in Fig. 1. The percentage of drug released from c-SLN was plotted as a function of time. The release of the drug from nanoparticles prepared using stearic acid as lipid was conducted over a period of 96 h. As shown in Fig. 1a, the release of FA in PBS solution was faster compared to ASP at initial time point up to 12 h. A cumulative drug release of approximately 98 % of ASP was observed within 48 h of the study, and then showed constant release up to 96 h. The FA c-SLN showed approximately 98 % drug release at 72 h, even though it showed a faster release pattern in initial time points compared to ASP. Both the formulations exhibited slow sustained release of the drug. The release profile was similar in acidic condition at pH 1.6 compared to PBS solution at pH 6.8 (Fig. 1b).

### Determination of IC_50_ concentration of free and c-SLN encapsulated FA and ASP

**Fig. 2:**
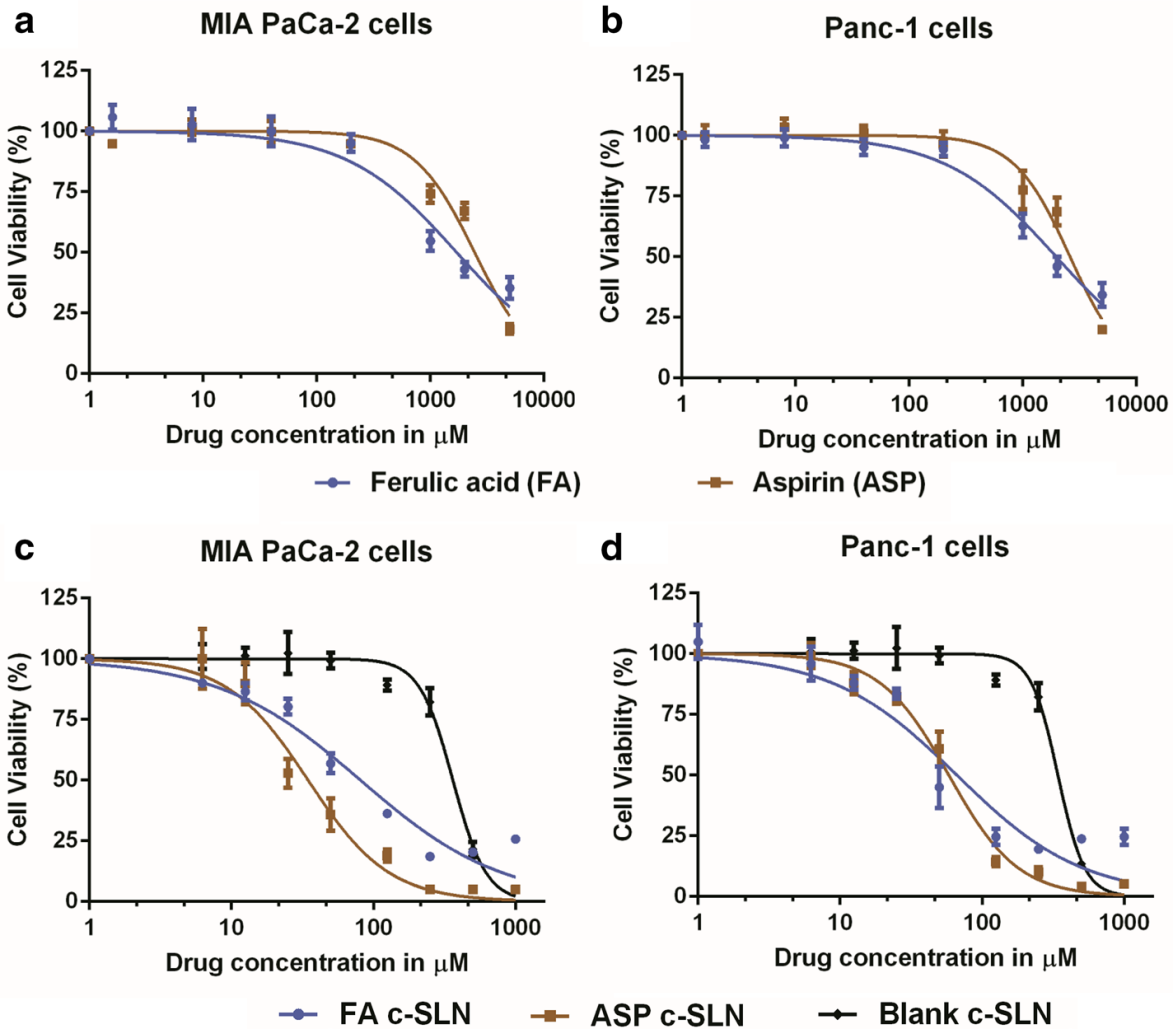
Dose dependent inhibition of cell viability in pancreatic cancer cells. **a**, **c** MIA PaCa-2 and **b**, **d** Panc-1 cells were treated with unmodified ferulic acid (FA), unmodified aspirin (ASP), FA encapsulated in chitosan-solid lipid nanoparticles (c-SLNs), and ASP c-SLNs. MTS assay was performed to determine the cell viability of MIA PaCa-2 and Panc-1 cells after treating with a range of concentrations of free and c-SLN modified FA and ASP for 72 h. The IC_50_ values were then determined using nonlinear regression using graph pad prism software. Each data point represents the mean percent viable cells measured in three parallel but independent experiments

In order to evaluate the combined effects of FA and ASP on pancreatic cancer, we first treated MIA PaCa-2 and Panc-1 cells with various individual concentrations (5–5000 µM) of FA and ASP for 72 h, and cell growth was measured by the MTS assay. Our observations show that there was a dose-dependent inhibition of the growth of MIA PaCa-2 and Panc-1 cells. In case of MIA PaCa-2, the IC_50_ values for FA and ASP observed were 1.7 and 2.6 mM, respectively (Fig. 2a); whereas in Panc-1 cells, the IC_50_ values for FA and ASP were 1.9 and 2.4 mM, respectively (Fig. 2b).

As shown in Fig. 2, cell viability assay was performed with serial dilutions (1–1000 µM) of FA and ASP c-SLN nanoparticle formulations. In MIA PaCa-2 cells, the IC_50_ concentrations for FA c-SLN, ASP c-SLN, and blank c-SLN observed were 81.15, 34.66, and 362 μM, respectively (Fig. 2c); whereas in Panc-1 cells, the IC_50_ values for FA c-SLN, ASP c-SLN, and blank c-SLN were 66.6, 58.32, and 341.8 µM respectively (Fig. 2d).

### Effect of the combination of FA and ASP c-SLN on pancreatic cancer cells

**Fig. 3:**
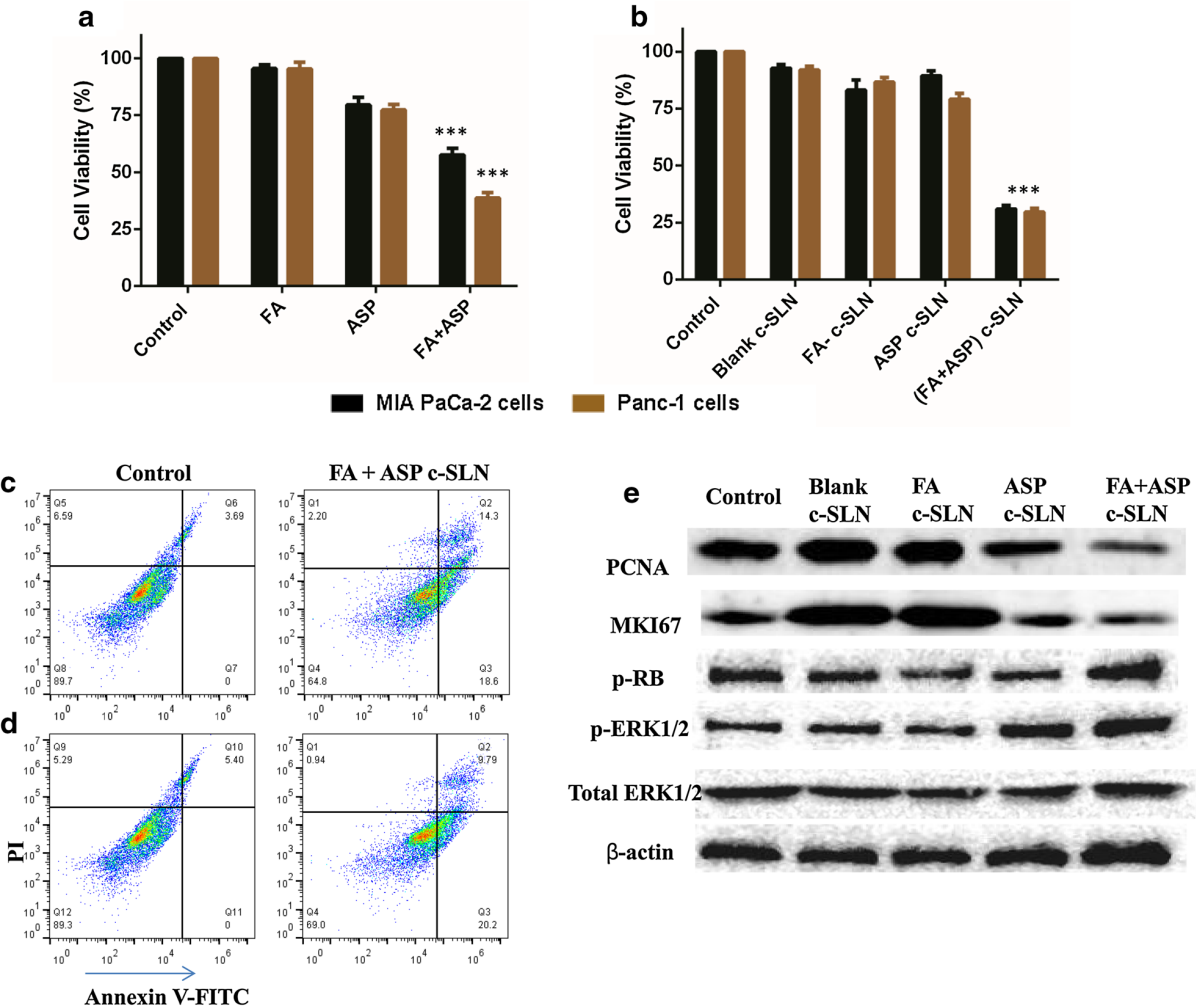
Synergistic effect of ferulic acid (FA) and aspirin (ASP) combination on cell viability and apoptosis. MTS assay was performed to determine the cell viability of **a** free FA + ASP and **b** chitosan-solid lipid nanoparticle (c-SLN) encapsulated FA + ASP combination. MIA PaCa-2 and Panc-1 cells were treated with free FA (200 µM), with ASP (1 mM) and c-SLN FA (40 µM) with c-SLN ASP (25 µM) individually and in combination for 72 h. Each *bar* represents the mean percent viable cells measured in three parallel but independent experiments. **c** MIA PaCa-2 and **d** Panc-1 cells were treated with c-SLN encapsulated FA (40 µM) with c-SLN ASP (25 µM) for 48 h and stained with Annexin V-PI apoptosis detection kit. **e** Panc-1 cells were treated with FA (40 µM) with c-SLN ASP (25 µM) alone or in combination for 24 h. The expression of PCNA, MKI67, phospho-RB, and phospho-ERK1/2 proteins were evaluated by western blot analysis. The equal loading was confirmed by using an anti-β-actin antibody. Statistical significance was determined by one-way ANOVA followed by Dunnett’s multiple comparison test post hoc *analysis.* ***P < 0.001 represents statistical significance of differences between control and treatment group

To examine the effect of combined regimen on cell proliferation, MIA PaCa-2 and Panc-1 cells were treated with low and ineffective concentrations of free form FA (200 μM) and ASP (1 mM) for 72 h. As shown in Fig. 3a, single agents did not show significant change in cell viability at these concentrations. However, when used in combination at identical concentrations, FA + ASP showed a significant effect with a reduction in cell viability of MIA PaCa-2 cells by as much as 45 % (P < 0.01), whereas FA + ASP combination in Panc-1 cells showed a remarkable decrease of 60 % (P < 0.001) cell viability.

In case of c-SLNs, after determining the dose response curves individually and obtaining the IC_50_ value for FA and ASP c-SLNs, ineffective and low concentrations were selected for FA c-SLN (40 µM**)** and ASP c-SLN (25 µM) showing minimal inhibitory response on the cell lines (Fig. 3b). When combined together (FA + ASP c-SLNs), the cell viability was reduced to 70 % for MIA PaCa-2 and Panc-1 cells, respectively (P < 0.001). Thus, the combination of FA and ASP c-SLN administered at low concentrations showed a significant reduction in cell viability compared to unmodified, free form combinations of FA and ASP. The blank c-SLN at same concentration did not show reduction in cell viability, hence demonstrating that c-SLN formulation excipient has no significant effect on cell viability of pancreatic cancer cells (Fig. 3b).

### FA and ASP combination induces apoptosis in pancreatic cancer cells

The induction of apoptosis was measured by flow cytometry for all individual drugs and their combinations on MIA PaCa-2 and Panc-1 cells. Individual concentrations of FA c-SLN (40 µM) and ASP c-SLN (25 µM) showed minimal apoptotic cells (data not shown). In case of MIA PaCa-2 cells (Fig. 3c), FA and ASP c-SLN combinations demonstrated approximately 35 % apoptotic cells (P < 0.01). In case of Panc-1 cells (Fig. 3d), c-SLN combined FA + ASP showed 31 % of apoptotic cells (P < 0.01). Overall, our studies confirmed that FA + ASP combinations were significantly effective in inducing apoptosis of cancer cells.

### FA and ASP combination modulates proliferation proteins in pancreatic cancer cells

From our previously published studies, aspirin was reported to modulate proliferation proteins and ERK pathway [6, 7], hence we wanted to determine whether the proliferation proteins like PCNA, MKI67, RB and ERK1/2 are involved in growth inhibition. The MIA PaCa-2 and Panc-1 protein lysates were analyzed by western blot analysis. We observed that incubation of MIA PaCa-2 and Panc-1 cells with combination FA + ASP inhibits PCNA and MKI67 proteins, and produced higher phosphorylation of ERK1/2 and RB proteins compared with the control and individual drugs (Fig. 3e).

### FA and ASP c-SLN in a pancreatic xenograft tumor model

**Fig. 4:**
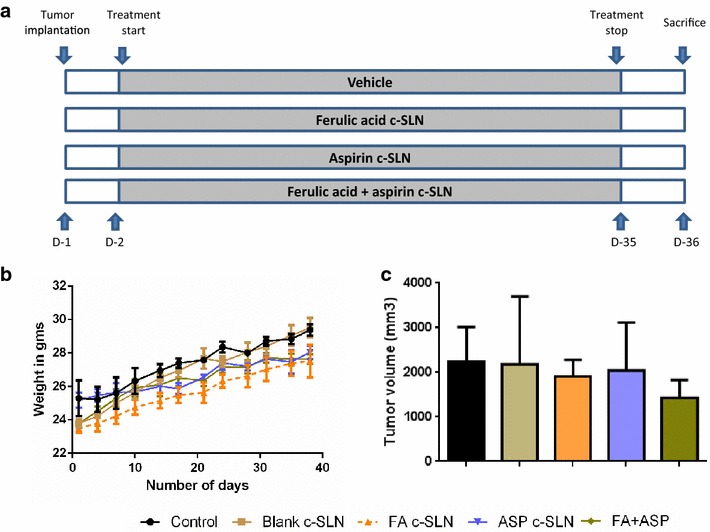
In vivo effect of ferulic acid (FA) and aspirin (ASP) c-SLNs on MIA PaCa-2 xenograft SCID mice. **a** Flow chart representation of experimental design for in vivo studies. **b** Body weight variations of xenograft SCID mice during the FA and ASP c-SLNs treatment period. Average body weight transitions of mice over the 35 days treatment period. **c** Average tumor growth of pancreatic cancer cells MIA PaCa-2 over a period of 35 d after treatment. FA and ASP c-SLNs were administered via oral gavage for 35 days. Statistical significance was determined by one-way ANOVA followed by Dunnett’s multiple comparison test post hoc *analysis*

In vivo studies using a xenograft mouse model was subsequently conducted to determine whether oral administration of FA + ASP c-SLN combinations could suppress the growth of pancreatic tumor. MIA PaCa-2 cells were implanted subcutaneously in SCID mice. The treatment by oral gavage started 1 day after tumor implantation and continued as per protocol for 5 weeks (Fig. 4a). The FA and ASP c-SLN were administered at the dose of 75 and 25 mg/kg daily via oral gavage. During the experiment, all mice were monitored to investigate possible adverse effects caused by drug treatment. The FA and ASP c-SLN treatment did not cause any significant weight loss of the mice (Fig. 4b). These results suggested that no noticeable side effects or toxicity were caused by the treatment regimen. Furthermore, no gross signs of toxicity were observed during visible inspections of general appearance and macroscopic examinations of individual organs.

In an effort to establish the efficacy of a combined therapy compared with single-agent treatment, we determined the mean tumor volume in all treated groups. For instance, at day 36 the mean tumor volume (mean ± SEM) in control and blank c-SLN (vehicle control) mice were 2576 ± 530 and 2513 ± 352 mm^3^ respectively, as compared with 2235 ± 244 mm^3^ in FA c-SLN and 2713 ± 736 mm^3^ in ASP c-SLN treated mice. However, mean tumor volume in FA + ASP c-SLN group was 1424 ± 402 mm^3^ (Fig. 4c). The administration of FA and ASP c-SLN combination treatment resulted in 45 % reduction in the mean tumor volume compared with vehicle control group, though this reduction was statistically non-significant.

### FA and ASP inhibits expression of proliferation markers in pancreatic tumor tissues

**Fig. 5:**
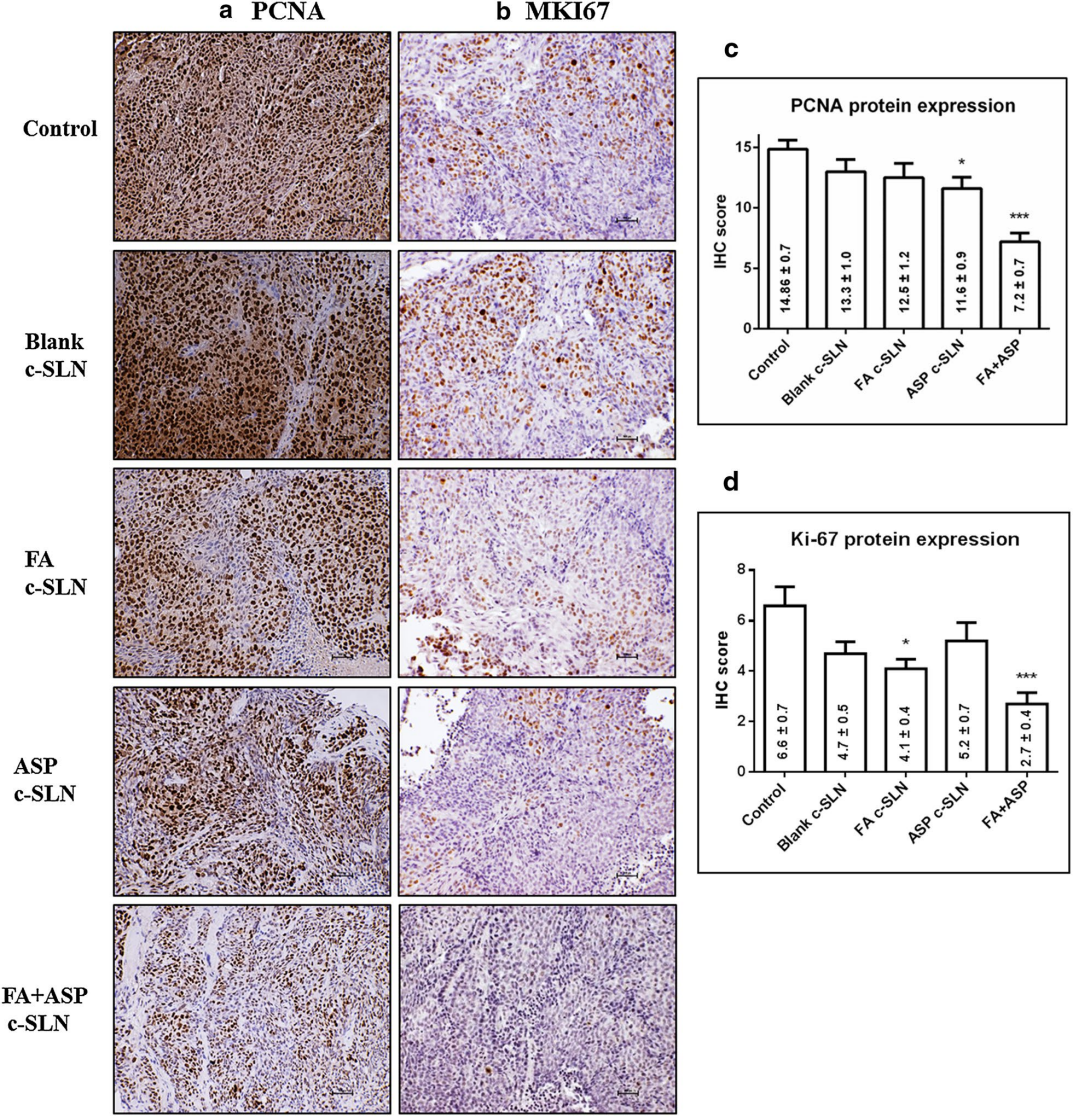
Effect of ferulic acid (FA) and aspirin (ASP) chemopreventive regimen on tumor cell proliferation and apoptosis. Immunohistochemical analysis was performed on paraffin-embedded and micro-sectioned pancreatic tissues as described in methods section. **a**, **b** Representative images showing effect of modified FA and ASP c-SLN combination on PCNA and MKI67 (marker of proliferation Ki-67) expressions in pancreatic tissue. **c**, **d** A significant decrease in the PCNA and MKI67 expression was observed in modified FA + ASP treatment groups compared to blank c-SLN vehicle control group. All the pictures were taken at ×200 magnification. Statistical significance was determined by one-way ANOVA followed by Dunnett’s multiple comparison test post hoc *analysis.* *p < 0.05; ***p < 0.001 represents statistical significance between blank c-SLN vehicle control and FA + ASP c-SLN treatment

In order to evaluate the effect of FA + ASP chemopreventive regimens on tumor cell proliferation, immunohistochemistry (IHC) was performed to measure the expression of cell proliferation markers proliferating cell nuclear antigen (PCNA; Fig. 5a, c) and MKI67 (Fig. 5b, d) on pancreatic tissues. The protein expression was evaluated by IHC scores as described in the methods section. The results in Fig. 5 showed that FA c-SLN in combination with ASP c-SLN showed 46 and 43 % down-regulation in the expression of PCNA and MKI67 proliferation proteins respectively in tumor tissues compared with the blank c-SLN control group (p < 0.001). Together, our results suggest that FA and ASP c-SLN combination treatment down regulates expression of proliferation proteins.

### FA and ASP c-SLN combination inhibits apoptotic and cell cycle proteins in pancreatic tumor tissues

**Fig. 6:**
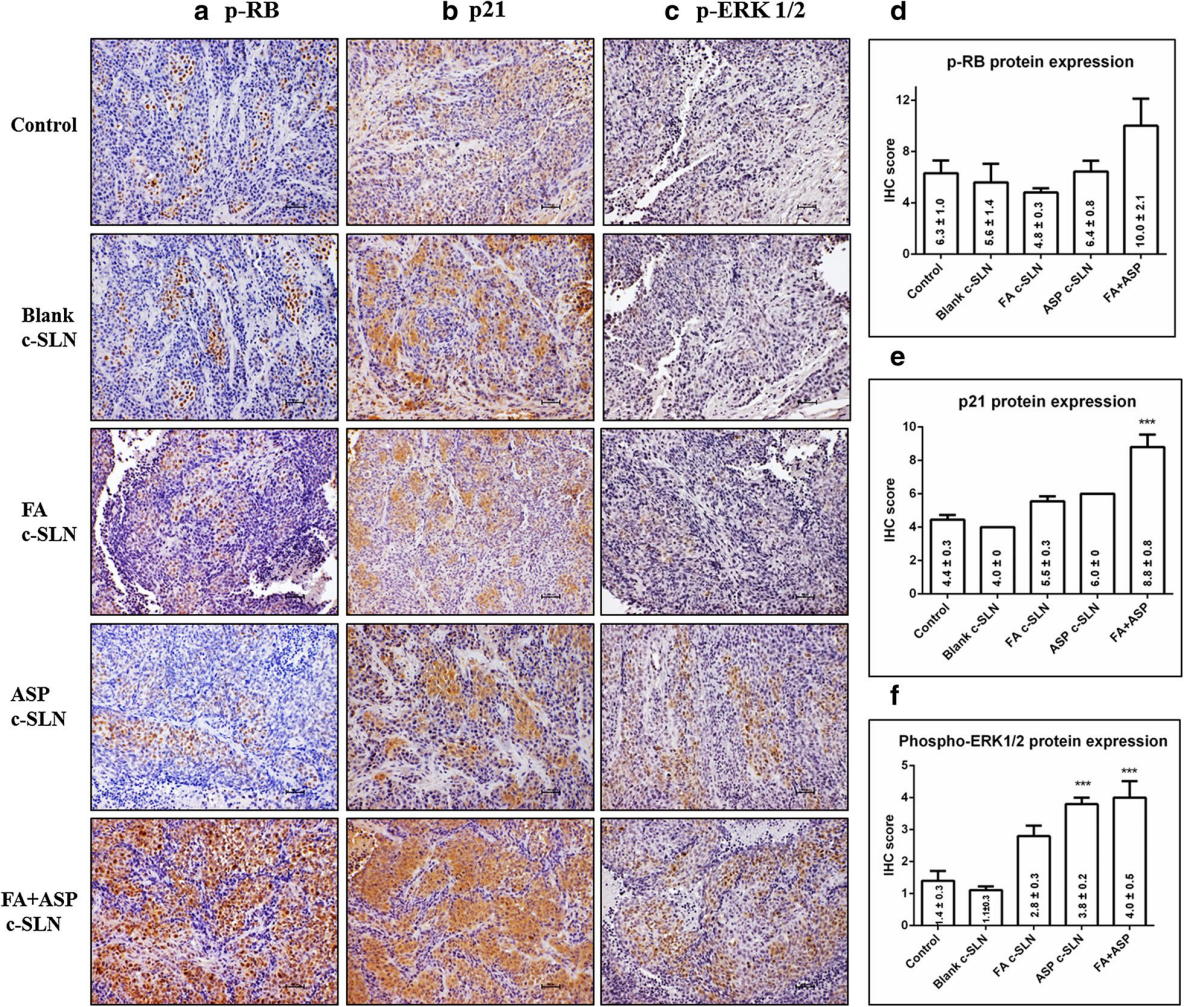
Effect of ferulic acid (FA) and aspirin (ASP) chemopreventive regimen on apoptotic proteins. Immunohistochemical analysis was performed on paraffin-embedded and micro-sectioned pancreatic tissues as described in methods section. **a**–**c** Representative images showing effect of modified FA and ASP c-SLN combination on p-RB, p21, and p-ERK1/2 protein expressions in pancreatic tissue, respectively. **d**–**f** An increase in the p-RB, p21 and p-ERK1/2 protein expressions was observed in FA + ASP c-SLN treated groups compared to the vehicle control group. All the pictures were taken at ×200 magnification. Statistical significance was determined by one-way ANOVA followed by Dunnett’s multiple comparison test post hoc *analysis.* ***p < 0.001 represents statistical significance between blank c-SLN vehicle control and FA + ASP c-SLN treatment

Important families of proteins that are involved in the regulation of apoptosis are mammalian mitogen activated protein kinases (MAPK) that can be subdivided in extracellular signal-regulated kinases (ERKs) that regulate cell growth and differentiation [30, 31]. Also anti-carcinogenic effect of FA has been linked to down-regulation of an anti-apoptotic p21 protein [32]. Based on this information, we evaluated the expression of phosphorylated retinoblastoma (p-RB) protein, phospho-ERK1/2 and p21 protein expression using IHC. As shown in Fig. 6, the FA + ASP c-SLN combination group showed 220 and 178 % up-regulation of p21 and p-RB proteins compared to vehicle control, which are anti-apoptotic and tumor suppressor protein, respectively. The FA + ASP c-SLN treatment group also showed 363 % upregulation of p-ERK1/2 protein compared to vehicle control, which correlated with our published chemoprevention studies of ASP combinations, suggests activation of p-ERK1/2 as a mechanism of action of ASP [6].

## Discussion

Pancreatic carcinoma arises from a heterogeneous molecular pathogenesis involving several oncogenic pathways and defined genetic mutations. Studies have suggested that chronic oxidative stress, particularly from chronic inflammation, is associated with carcinogenesis [33, 34]. For example, ulcerative colitis has long been linked with high incidence of colorectal cancer; and chronic gastrititis, such as from infection with *H. pylori*, has been associated with a high incidence of gastric cancer [35, 36]. Recent studies have shown an important role for ROS in tumor development [37, 38]. ROS can be produced from endogenous sources, such as from mitochondria, peroxisomes, and inflammatory cell activation [34, 39]; and exogenous sources, including environmental agents. This oxidative stress then, in turn, may cause DNA, protein, and/or lipid damage, leading to changes in chromosome instability, genetic mutation, and/or modulation of cell growth that may result in cancer. The potential outcomes of oxidative stress occur when not counter-balanced by antioxidant defenses of the cell.

Previous studies in literature show the uptake of SLNs from the intestinal lymphatic system thus bypassing first pass metabolism in the liver, increasing circulation time, reducing dosage and ensuring high bioavailability of the drugs [40–43]. The addition of chitosan also provides steric stabilization of nanoparticles thus reducing their uptake by the reticulo-endothelial (RES) system in the blood [44, 45]. Moreover, chitosan can mediate the opening of tight junctions between epithelial cells reversibly, facilitating the paracellular transport of drug molecules ultimately leading to improved bioavailability of the drugs [29]. Thus, c-SLN combines the advantages of SLN with the biological properties of chitosan as a drug delivery vehicle.

The effect of FA and ASP was initially evaluated by calculating the IC_50_ values and then by combining the ineffective concentrations to exhibit an additive or synergistic effect against the pancreatic cancer cells proving to be more efficacious at lower concentrations. When free FA and ASP were combined at ineffective concentrations of 200 μM and 1 mM respectively, showed 45–60 % inhibition of cell growth. The cell viability assay on FA and ASP entrapped c-SLNs was carried out using FA (40 µM) and ASP (25 µM) as individual concentrations. Individually, they showed little or no decrease in the cell viability, but when combined, a significant reduction by 70 % was observed in MIA PaCa-2 and Panc-1 cells. However, a comparative study between the two forms of the drugs i.e., the free form and c-SLN form showed approximately 5 and 40-fold reductions in FA c-SLN and ASP c-SLN respectively in comparison to the free form of the drugs. Studies have been reported where drug loaded c-SLNs have exhibited better cytotoxicity profile in comparison to the free drug [3, 9]. This has been mainly attributed to the smaller particle size of the nanoparticles which increases the overall uptake of the drug. In order to validate the efficacy of the combination regimen, apoptosis assay was conducted which determined the progression of a cancer cell from four different phases after the addition of the drug: living cell, early apoptotic cell, late apoptotic cell and necrotic cells. These results are consistent with our findings in the cell viability assay.

Pancreatic tumor growth inhibition in in vivo xenograft mice model was observed by the oral administration of 75 and 25 mg/kg FA and ASP c-SLN combination, respectively, even though the effect was not statistically significant. In our previously published study [7], we used SLN encapsulated aspirin at a dose range of 2–20 mg/kg, which demonstrated to be effective in suppressing the progression to pancreatic cancer in a hamster model. In this study, we used a conversion factor to calculate the dose given to the hamster to mouse which resulted in 25 mg/kg of ASP for the current study [46]. For FA, dose of 75 mg/kg used for these experiments were deduced from publications demonstrating the use of higher doses than the proposed dose for the current studies [19, 47, 48]. Other in vivo studies have found elevated levels of detoxifying enzymes in liver and colonic mucosa in rats fed with FA at the dose of 100 mg/kg [17]. In the same study, FA reduced the incidence and multiplicity of intestinal azoxymethane-induced tumors after 35 weeks of oral administration of 250 mg FA/kg body weight/day.

IHC was performed to evaluate the molecular effect of FA + ASP chemopreventive regimens on tumor reduction. This study showed decreased proliferation as documented by PCNA and MKI67 immunostaining, and increased apoptosis as documented by increased p21 expression within tumors. Moreover, our results demonstrate that the upregulation of p-ERK1/2 and p-RB proteins in FA + ASP treatment group compared to vehicle control group. A number of studies have shown the importance of ERK signaling pathway in regulating apoptosis [6, 49, 50]. Although ERK pathway delivers a survival signal, recent studies and our previously published article on chemoprevention of aspirin have linked the activation of p-ERK with induction of apoptosis [51–53]. Our published study also presents a plausible mechanism by which aspirin, curcumin, sulforaphane (ACS) in combination can induce apoptosis in pancreatic cancer cells through activation of the p-ERK1/2 signaling system. The ACS combination initiated p-ERK1/2 induction at 8 h, and the activity remained highly elevated through the remaining time period examined (48 h). It is important to note that there are different mechanisms of ERK activation, such as induction by growth factors could be rapid (occurring within minutes) and transient, which leads to cell proliferation and survival [54]. But persistent or sustained p-ERK1/2 activation that lasts more than 12 h is involved in cell differentiation and death [55]. Our results support the pro-apoptotic role of p-ERK1/2 during FA + ASP treatment and are in agreement with previous studies [6, 51–53].

## Conclusions

This study investigated the potential chemopreventive effects of a combination of free FA and ASP as well as c-SLN encapsulated FA and ASP. We demonstrated for the first time that the FA and ASP c-SLN combination showed a synergistic inhibition of cell viability and induced apoptosis in MIA PaCa-2 and Panc-1 human pancreatic cancer cells. Further in vivo studies demonstrated the tumor shrinking capability of this c-SLN combination, even though this was not statistically significant. Immunohistochemical studies indicate the pro-apoptotic role of the regimen from significantly elevated protein expression. In conclusion, the preliminary data obtained from these studies provide a baseline on which further research is warranted to confirm this nanotechnology-based combination regimen as a potentially viable chemopreventive tool in the fight against pancreatic cancer.

## Methods

### Cell lines and cell culture

Human pancreatic cancer cell lines MIA PaCa-2 and Panc-1 were obtained from American Type Culture Collection (ATCC, Manassas, VA, USA). Cells were cultured in Dulbecco’s modified eagle medium (DMEM) supplemented with 10 % fetal bovine serum (FBS), and 1 % penicillin–streptomycin at 37 °C in a 5 % CO_2_ humidified environment.

### Reagents and antibodies

FA and ASP were purchased from Sigma-Aldrich (St. Louis, MO, USA). Stearic acid, Poloxamer 188, chitosan and lecithin was obtained from Spectrum Chemicals (Gardena, CA, USA). Dichloromethane (DCM) was obtained from Fisher Scientific (Houston, TX, USA). Sodium chloride was purchased from ChemCruz (Santa Cruz, CA, USA). Hydrochloric acid was purchased from Ricca Chemicals (Arlington, TX, USA). The primary antibodies against PCNA, p-ERK1/2 (Thr202/Tyr204), and p-RB were obtained from Cell Signaling Technologies (Danvers, MA, USA). The MKI67 and p21 primary antibodies were purchased from Abcam (Cambridge, MA, USA).

### Preparation of chitosan solid lipid nanoparticles (c-SLNs)

**Fig. 7:**
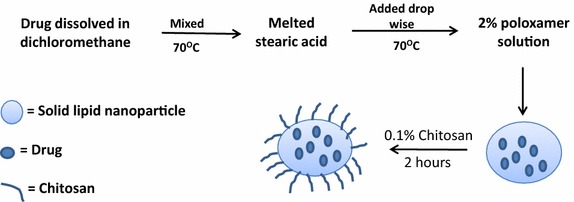
Schematic representation of chitosan solid lipid nanoparticle (c-SLN) formulation development

FA and ASP c-SLNs were prepared using a hot melt oil-in-water (o/w) emulsion technique (Fig. 7). Stearic acid was used as the lipid to make nanoparticle formulations. Briefly, 1 g of stearic acid was melted by heating at 70 °C. The drug (250 mg) was dissolved in 5 ml of DCM. The drug solution was then added to the melted stearic acid and heated until all DCM was evaporated. The water phase consisted of 2 % poloxamer solution which was heated to the same temperature as that of the oil phase. The volume ratio of oil phase to water phase was kept at 1:10. The oil phase was then added to the poloxamer solution drop wise using continuous high sheer homogenization and the mixture was further sonicated for 30 s using an ultra-sonicator (Los Angeles, CA, USA) to create an o/w emulsion. The emulsion so formed was mixed with equal volume of 0.1 % chitosan dissolved in 0.1 % acetic acid. The mixture of SLNs with chitosan was stirred for 2 h. The resulting chitosan-SLNs were centrifuged and pellet was collected. The c-SLNs were lyophilized in a freeze dryer (Labconco, Kansas City, MO, USA) and subjected to particle size and encapsulation efficiency determination.

### Measurement of particle size, encapsulation efficiency and zeta potential

The mean particle size (z-average) and polydispersity index (PDI) as a measure of the width of particle size and distribution was determined by photon correlation spectroscopy using Zetasizer (Nano ZS 90, Malvern Instruments, Malvern, UK) at 25 °C and 90° scattering angle. The c-SLN formulation was diluted with nano-pure water to weaken opalescence before measurements. The surface charge was assessed by measuring zeta potential of c-SLNs based on the Smoluchowski equation, using the same equipment at 25 °C with electric field strength of 23 V/cm [54].

### Determination of percentage encapsulation efficiency of FA and ASP c-SLNs

Encapsulation efficiency was determined by dissolving 10 mg of the c-SLN formulation in 10 ml acetonitrile. The drug was released from the lipid into acetonitrile and allowed to dissolve freely for 10 min in a sonicator after which it was filtered through a 0.45 µm filter. The resulting solution was further diluted with acetonitrile and was analyzed by using a Shimadzu LC-20 binary HPLC system (Columbia, MD, USA). Caffeine was used as the internal standard. The entrapment efficiency was calculated using the following formula:

$$EE \, \left( \% \right) \, = \, \left[ {Amount \, \left( {mg} \right) \, of \, drug \, per \, HPLC \, method \, / \, theoretical \, yield \, \left( {mg} \right)} \right] \, \times \, 100.$$

### In vitro drug release from FA and ASP c-SLNs

The cumulative release of ASP and CUR from c-SLNs was determined in both phosphate buffered saline (PBS), pH 6.8 and acidic medium, pH 1.6. Briefly, acidic medium composed of a mixture of sodium chloride (34.2 mM), lecithin (20 µM) and hydrochloric acid with pH adjusted to 1.6. Five mg of the c-SLNs were suspended in 50 ml of PBS/acidic medium and placed in an incubator at 37 °C with a shaking speed of 100 rpm. At predetermined time intervals (0, 0.5, 1, 2, 4, 6, 12, 24, 48, 72, 96 h), 1 ml of the buffer was withdrawn and replaced with equivalent volume of fresh buffer. All samples are centrifuged at 5000 rpm for 10 min. The amount of released drug was analyzed using HPLC. The analysis was carried out in triplicate.

### Cell viability assay

The cell viability assay was performed according to the manual included with the Promega Cell Titre 96 Aqueous MTS reagent (Madison, WI, USA). Briefly, 7.5 × 10^3^ cells were seeded in 96 well plates and treated with FA and ASP alone and in combination for a period of 72 h. On the last day of the incubation period, 20 % MTS and 1 % of phenazine methosulfate (PMS) were added to the medium and incubated for 2 h at 37 °C and absorbance was measured at 490 nm. All the assays were performed in triplicate.

### Flow cytometric analysis for apoptosis

The detection was performed according to the manual included with the Annexin V-fluorescein isothiocyanate (FITC) Vybrant Apoptosis assay kit #3 (Invitrogen, Grand Island, NY). Approximately 1 × 10^5^ MIA PaCa-2 and Panc-1 cells were seeded in six-well plates and treated with c-SLN modified FA and ASP alone and in combination at concentrations of 40 and 25 μM, respectively. After incubation for 48 h, cells were harvested, washed twice with ice-cold phosphate buffer saline (PBS), and then subjected to 5 µl of FITC Annexin V and 1 µl of the 100 µg/ml PI. The samples were analyzed using Beckman Coulter Cytomics FC500.

### Western blot analysis

MIA PaCa-2 and Panc-1 cells were treated with FA and ASP alone and in combination for 24 h. Cells were lysed in RIPA buffer and were fractionated on SDS–PAGE gels and then transferred to nitrocellulose membranes. The membranes were blocked with 2 % bovine serum albumin (BSA) in tris-buffered saline (TBS)-Tween 20 and probed with primary antibodies (1:1000 dilution) followed by horseradish peroxidase (HRP)–labeled secondary antibodies (1:5000 dilutions). The blots were probed with the Super Signal West Pico Chemiluminescent substrate (Thermo Scientific, Pittsburgh, PA, USA) to visualize the immunoreactive bands.

### Tumor xenograft model

The study was conducted on male severe combined immunodeficient (SCID) mice, 6 weeks old with an average body weight of ~20 g. All animals were maintained in pathogen-free sterile isolators and in a controlled atmosphere with a 12 h light to 12 h dark cycle according to institutional guidelines. All studies were conducted as per protocol approved by the Western University of Health Sciences Institutional Animal Care and Use Committee and conformed to the “Principles of Laboratory Animal Care”. Mice were gavage daily with the chemopreventive regimen and weighed twice a week throughout the experimental period. The human pancreatic cancer cells MIA PaCa-2 were grown in DMEM media containing 10 % FBS and harvested in HyQTase cell detachment solution (Hyclone). Cells were resuspended in saline at 1 × 10^6^ cells/0.1 ml volume, and placed on ice. SCID mice were injected with 0.1 ml of the cell suspension subcutaneous on the right flank and observed daily for tumor growth. After transplantation, tumor size was measured using calipers, and the tumor volume was estimated according to the following formula: tumor volume (mm^3^) = L × W^2^, where L is the length and W is the width. After 5 weeks, mice were sacrificed and tumors were excised immediately.

### FA + ASP c-SLN treatment regimen

As indicated in Table 2, this set consisted of five groups (T1–T5) of 4 mice per group. The treatment groups T3-T5 received FA c-SLN, ASP c-SLN alone and a combination of FA + ASP c-SLN regimen daily via oral gavage, respectively. Treatment started the day after tumor cell implantation and continued every 24 h for 5 weeks. The T1 group served as control and received 0.1 ml PBS, and T2 group received blank c-SLN serving as vehicle control. The dose selected for FA c-SLN and ASP c-SLN regimens were 75 and 25 mg/kg respectively, which was determined based on current evidence in literature [19, 48, 55].

**Table 2:** Treatment plan showing group of mice treated with c-SLN modified FA, and ASP

Groups	Number of mice	Treatment plan	Dose (mg/kg)
T1	4	Saline (control)	0
T2	4	Blank c-SLN (vehicle control; no drug)	100
T3	4	FA c-SLN	75
T4	4	ASP c-SLN	25
T5	4	FA + ASP c-SLN combination	75 + 25

*ASP* aspirin, *FA* ferulic acid, *c-SLN* chitosan solid lipid nanoparticle

### Histological examination

All organs of the thoracic and abdominal cavities were carefully examined in situ macroscopically after euthanization. The MIA PaCa-2 tumor tissue was fixed in 10 % phosphate-buffered formalin for 24 h. The formalin-fixed pancreatic tumor was cut into small pieces at 2 cm intervals, and 5 μm thick sections were processed.

### Immunohistochemistry (IHC)

Paraffin-embedded sections of pancreatic tumor tissue were deparaffinized, rehydrated, and heated in citrate buffer (pH 6.0) for 20 min for antigen retrieval. Endogenous peroxidase activity was quenched by incubating the slides in 3 % hydrogen peroxide, followed by washing in PBS-Tween 20. Subsequently, 5 % normal goat serum blocking buffer was applied. The blocking buffer was removed after 1 h of incubation in humidified chamber and primary antibody was added to the slides, incubated overnight at 4 °C. The horseradish peroxidase (HRP)-labelled secondary antibody (Cell Signaling Technologies) was then added and incubated for 90 min at room temperature. The section was developed with ImmPACT DAB peroxidase substrate kit (Vector Labs, Burlingame, CA, USA).

### Evaluation of staining

Antibody stained tissues were assessed using scoring system based on the Quickscore method [56]. For IHC staining, the protein stained in brown color was considered labeled/positive and nuclear staining in blue as unlabeled/negative staining. Briefly, the proportion of positive cells were estimated and given a score on a scale of 1–4 (1 = 0–10 %; 2 = 11–30 %; 3 = 31–59 %; 4 = 60–100 %). The intensity of the staining was estimated and given a score from 1–4 (1 = no staining; 2 = weak; 3 = intermediate; and 4 = strong staining). A score was then calculated by multiplying the percentage of positive cells score by the intensity score, to yield a minimum value of 1 and a maximum value of 16.

### Statistical analysis

Results were expressed as mean ± SEM. A one-way ANOVA followed by Dunnett’s multiple comparison test post hoc *analysis* using Graphpad prism software (La Jolla, CA, USA) was done to analyze and compare the results. A probability value of ≤0.05 % was considered significant.

## Abbreviations

FA: ferulic acid
ASP: aspirin
c-SLN: chitosan-solid-lipid nanoparticle
NSAIDs: non-steroidal anti-inflammatory drugs
ROS: reactive oxygen species
DCM: dichloromethane
PDI: polydispersity index
PMS: phenazine methosulfate
FITC: fluorescein isothiocyanate
PCNA: proliferation cell nuclear antigen
ERK1/2: extracellular signal-regulated kinases
RB: retinoblastoma
IHC: immunohistochemistry
HRP: horseradish peroxidase
RES: reticuloendothelial system
MAPK: mitogen activated protein kinases
